# Percolation-tunable conductive MXene–hydrogel multi-channel conduits promote integrin-mediated peripheral nerve regeneration

**DOI:** 10.1016/j.mtbio.2026.103822

**Published:** 2026-11-07

**Authors:** Fang Liu, Yixuan Zhao, Yumeng Ren, Chunzhen Jiang, Mengjie Xu, Yifan Li, Yingxin Wei, Xiuyu Wen, Zongmei Ding, Feng Li, Luzhong Zhang, Yumin Yang, Jianghong He

**Affiliations:** a Jiangsu Key Laboratory of Tissue Engineering and Neuroregeneration, Key Laboratory of Neuroregeneration of Ministry of Education, Co-innovation Center of Neuroregeneration, NMPA Key Laboratory for Research and Evaluation of Tissue Engineering Technology Products, Nantong University, Nantong, 226001, China; b Fuyang People's Hospital, Fuyang, Anhui, 236000, China; c Department of Pathology, Northern Jiangsu People's Hospital Affiliated to Yangzhou University, Clinical Medical College, Yangzhou University, China; d Laboratory Medicine Center, The Sixth People's Hospital of Nantong (Affiliated Nantong Hospital of Shanghai University), Nantong, Jiangsu Province, 226001, China

**Keywords:** MXene, Hydrogel, Percolation conductivity, Multi-channel conduit, Peripheral nerve regeneration, Integrin-mediated mechanotransduction

## Abstract

Peripheral nerve repair is severely hindered by the lack of biomimetic conductive scaffolds that recapitulate native conductive microenvironments to coordinate neuronal differentiation and myelination. Here, we fabricate a multi-channel polyacrylamide/chitosan/MXene hydrogel nerve conduit with concentration-programmable percolative conductivity. A critical percolation threshold at 600 μg/mL establishes an optimal conductive microenvironment, which significantly enhances Schwann cell viability, neurite outgrowth, and neurotrophic factor secretion. Mechanistically, the MXene-mediated conductive cue upregulates myelination- and neuronal differentiation-related genes via activating integrin-dependent PI3K-Akt mechanotransduction and axon guidance signaling pathways. In a rat sciatic nerve defect model, the optimized conduit significantly promotes axonal regeneration and remyelination, with 12-week functional and electrophysiological recovery indicators showing no significant statistical difference from those of autologous nerve grafts. This work demonstrates that percolation-tunable conductive engineering is a reliable strategy to construct biomimetic neural microenvironments, providing a feasible translational scaffold for advanced peripheral nerve regeneration.

## Graphical abstract

**Figure:**
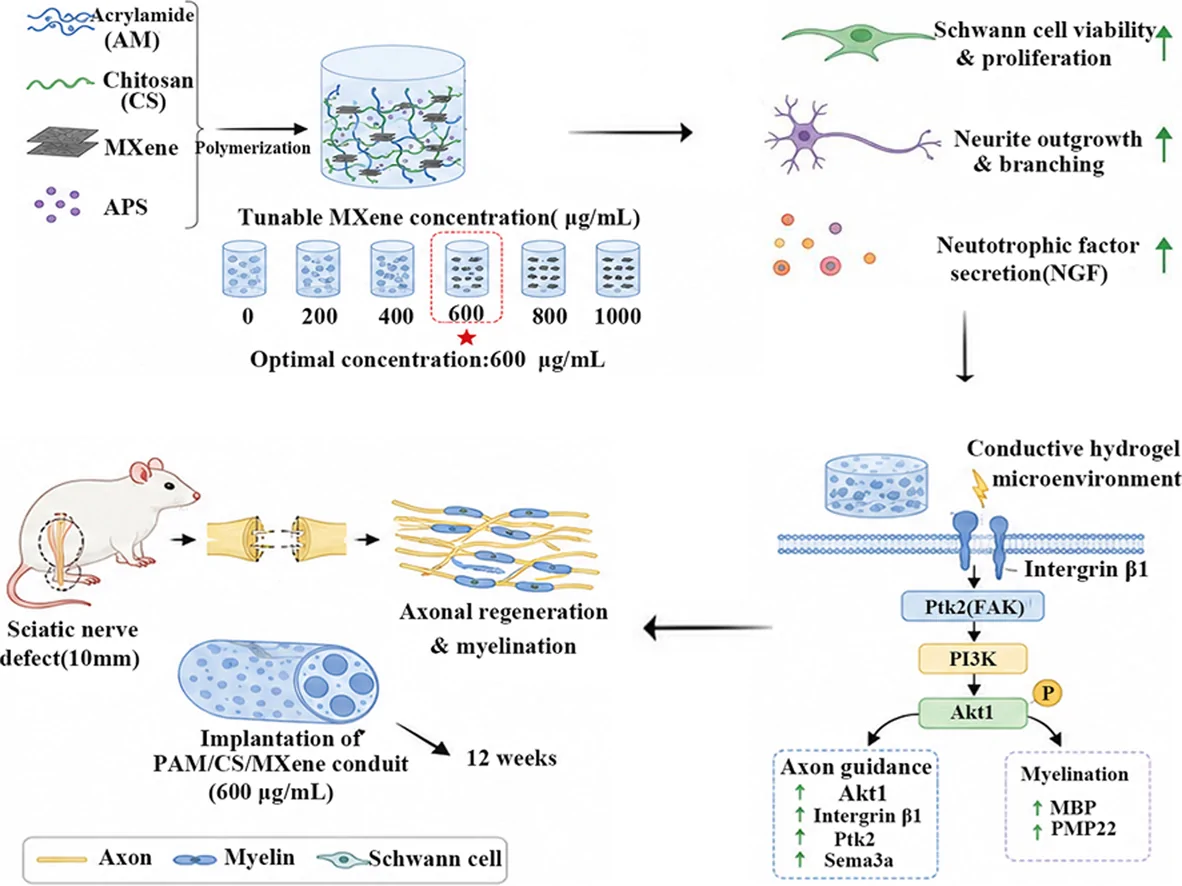

## 1 Introduction

Peripheral nerve injury (PNI) is one of the most common traumatic conditions in clinical practice, typically arising from mechanical trauma, crush injuries, lacerations, or iatrogenic surgical damage [1], [2]. Each year, millions of patients worldwide suffer from PNI, which frequently leads to chronic sensory deficits, motor dysfunction, and debilitating neuropathic pain. The consequences extend well beyond individual suffering—they place a substantial socioeconomic burden on healthcare systems and society at large [1], [2]. The intrinsic regenerative capacity of the peripheral nervous system, while not negligible, hits a hard ceiling when nerve gaps exceed approximately 5–10 mm [3], [4]. Beyond this threshold, spontaneous regeneration becomes largely ineffective, and surgical intervention is usually unavoidable. At present, autologous nerve grafting remains the clinical gold standard for bridging such defects. The rationale is straightforward: autografts provide a native three-dimensional architecture complete with endoneurial tubes, basal lamina, and endogenous neurotrophic factors such as Nerve Growth Factor (NGF) and Brain-Derived Neurotrophic Factor (BDNF) [5], [6]. In essence, they offer a physicochemical and biochemical microenvironment that synthetic materials have struggled to fully replicate.

Yet the reality of autografting is far from ideal. Donor nerve availability is inherently limited—harvesting the sural nerve, for instance, inevitably sacrifices sensation at the donor site and risks complications like painful neuroma or hypertrophic scarring [7]. This approach inevitably creates a trade-off between different performance attributes. Moreover, size and structural mismatches between donor and recipient nerves are the rule rather than the exception. Mismatched diameter or fascicular pattern frequently triggers aberrant axonal sprouting, misdirection, and poor target reinnervation [8], [9]. Add to this the morbidity of a second surgical site—infection risk, prolonged anesthesia, and vascular compromise—and the limitations become impossible to ignore [7], [10]. Given these constraints, developing alternative strategies that can bypass the drawbacks of autografts while actively guiding nerve regeneration has become an urgent priority in regenerative medicine and tissue engineering.

In this context, nerve tissue engineering has stepped forward as a particularly attractive alternative [9]. The goal is no longer merely to build a passive physical conduit that axons can crawl through. Rather, the aim is to engineer scaffolds that actively recreate the multifaceted regenerative niche of native nerve tissue—offering topographical guidance, mechanical support, biochemical signals, and critically, biophysical cues [11], [12], [13]. Among these biophysical cues, the endogenous bioelectrical environment deserves special attention. Electrical signaling is, after all, the very language of the nervous system—action potentials and nodal currents are how neurons communicate [14]. The pioneering work of Hodgkin and Huxley laid the ionic foundation for understanding these electrical phenomena, revealing the extraordinary sensitivity of neural membranes to electrical stimuli [15]. What makes this particularly relevant for tissue engineering is that injury sites naturally generate endogenous electric fields (EFs) due to transepithelial potential differences. These EFs are not passive bystanders; they actively direct growth cone migration, Schwann cell recruitment, and overall cellular reorganization during regeneration [16]. Decades of experimental work have shown that applied electrical stimulation (ES) can markedly accelerate axonal outgrowth, improve Schwann cell alignment and proliferation, and promote remyelination, ultimately translating into faster functional recovery in both animal models and clinical trials [17], [18]. At the cellular level, ES triggers upregulation of neurotrophic factors like BDNF and GDNF, drives cytoskeletal reorganization, and activates pro-regenerative signaling cascades including PI3K/Akt and MAPK/ERK [19], [20]. It follows logically that equipping tissue engineering scaffolds with stable, tunable electroactivity—mimicking or even amplifying the body's native bioelectrical signals—represents a promising strategy for building “smart” constructs that can actively dialogue with neural cells [21], [22].

Motivated by this rationale, considerable effort has gone into developing electroconductive biomaterials for neural scaffold fabrication. To date, research has focused mainly on two fronts: conductive polymers such as polypyrrole (PPy), polyaniline (PANI), and poly(3,4-ethylenedioxythiophene) (PEDOT), and carbon-based nanomaterials including graphene and carbon nanotubes (CNTs) [23], [24]. here is no denying that these materials have pushed the field forward. They support neural cell adhesion, guide neurite extension, and enhance cellular responses under electrical stimulation, thanks largely to their adjustable conductivity [25], [26].

However, translating these materials to clinical peripheral nerve repair remains challenging. Conductive polymers, for one, suffer from poor biodegradability, mechanical brittleness in dry states or excessive softness when hydrated, and the risk of acidic oligomer or dopant leaching that can provoke unwanted inflammation [27], [28]. Carbon-based materials fare no better in some respects. Their near-non-biodegradability raises red flags for long-term biocompatibility and potential foreign body reactions. More worryingly, certain carbon nanotubes—due to their high aspect ratio and biopersistence—bear an uncomfortable resemblance to pathogenic fibers, with associated cytotoxicity concerns [29], [30]. Poor aqueous dispersibility and complex surface functionalization requirements further complicate their practical use. In short, no single material has yet managed to combine high electroconductivity, favorable biocompatibility, controlled biodegradation, and suitable mechanical properties in one package. This unmet need is precisely what drives the search for next-generation conductive biomaterials.

PAM has become a common synthetic hydrogel matrix for neural tissue engineering. Its appeal lies in facile in-situ polymerization, a highly tunable elastic modulus, and a homogeneous three-dimensional porous network [31], [32]. By adjusting the monomer and crosslinker fractions, one can readily match the soft mechanical range of native peripheral nerve tissue, while still allowing high loading of nanoscale conductive fillers and ionic additives; structural integrity is maintained under physiological conditions [33]. A major drawback, though, is that pure PAM is biologically inert. It lacks endogenous cell-adhesive ligands, which restricts Schwann cell attachment and neurite outgrowth [31].

Chitosan (CS) is a natural polysaccharide derived from chitin and can compensate for this biological limitation. It shows low immunogenicity and intrinsic bioactivity that supports Schwann cell adhesion and proliferation; it can also modulate the local inflammatory microenvironment after nerve injury [34], [35]. Pure chitosan hydrogels, however, usually have insufficient mechanical stability and poor structural maintenance under hydrated physiological conditions [33]. PAM and CS therefore complement each other: PAM provides a mechanically customizable, robust crosslinked scaffold, while CS introduces bioactive motifs for cell–matrix interactions, including integrin-related mechanotransduction. Such a PAM/CS dual matrix is also a useful platform for incorporating MXene nanosheets, which allows the construction of percolation-tunable conductive multi-channel hydrogel conduits for peripheral nerve regeneration.

Two-dimensional transition metal carbides and nitrides—typically produced by selective etching of MAX phases—have attracted intense interest as a new class of conductive nanomaterials. Ti_3_C_2_T_x_, the most widely studied member, exhibits metallic-grade electrical conductivity that is more than sufficient for building efficient conductive networks within composite scaffolds [36], [37]. Unlike hydrophobic carbon nanomaterials, MXene nanosheets present inherently hydrophilic surfaces rich in terminal functional groups (–OH, –O, –F). This surface chemistry matters: it ensures excellent dispersion in aqueous media and creates abundant active sites for strong interfacial bonding with polymer matrices such as polyacrylamide and chitosan, enabling homogeneous composite fabrication without the aggregation headaches common with CNTs or graphene [38], [39]. Mechanically, MXenes strike a useful balance between strength and flexibility, allowing them to reinforce hydrogel scaffolds without sacrificing the elasticity required for nerve tissue integration [40]. Early biocompatibility data look promising, and applications in biosensing, antimicrobial coatings, and drug delivery are already being explored [41], [42].

That said, the use of MXenes in neural tissue engineering—specifically for peripheral nerve regeneration—remains surprisingly thin on the ground. Most existing studies have focused on energy storage or catalysis, while fundamental questions about how MXene nanosheets interact with neural cells, how they modulate the electrophysiological microenvironment to steer Schwann cell myelination and neuronal differentiation, and how they perform in vivo remain largely unanswered. This knowledge gap is both a limitation and an opportunity. Against this backdrop, the present study sets out to develop a polyacrylamide/chitosan/MXene (PAM/CS/MXene) hydrogel conduit featuring a multi-channel architecture and concentration-programmable percolation conductivity. We hypothesized that by tuning MXene concentration across a percolation threshold, we could establish an optimal conductive microenvironment that selectively activates integrin-mediated mechanotransduction pathways, thereby coordinating Schwann cell myelination and neuronal differentiation to achieve functional nerve repair. Through systematic in vitro and in vivo characterization, we demonstrate that this approach not only overcomes the material limitations of earlier conductive scaffolds but also establishes a generalizable strategy for engineering conductive microenvironments in peripheral nerve regeneration.

## 2 Methods

### 2.1 Preparation of MXene

Dissolve 1.56 g of lithium fluoride (LiF) in 37.6 mL of hydrochloric acid (HCl) and stir for 30 min at 35°C to in situ generate a hydrofluoric acid (HF) etching system. Gradually add 1 g of Ti_3_AlC_2_ powder to this system in small, incremental portions, and continue stirring the mixture on a magnetic stirrer at 35°C for 4 days. Centrifuge to separate the solid and liquid phases. Wash the resulting precipitate with ultrapure water until the solution pH reaches 6, then dry it in an oven to obtain multilayer Ti_3_C_2_. Separately, dissolve 0.5 g of lithium chloride (LiCl) in 25 mL of ultrapure water and stir at 35°C for 30 min. Add 0.5 g of dried Ti_3_C_2_ powder and continue stirring at 35°C for 2 days. Perform a two-step centrifugation: first centrifuge at 3500 rpm for 5 min, then centrifuge at 3500 rpm for 1 h. Discard the supernatant and retain the precipitate. Dry the precipitate in an oven to obtain MXene.

### 2.2 Preparation of PAM/CS/MXene composite conductive hydrogels at different MXene concentrations

Add 1 g of MXene to 40 mL of ultrapure water and sonicate for 30 min to obtain a uniformly dispersed MXene solution with a concentration of 0.025 g/mL. Thoroughly rinse the gel plates with ultrapure water before assembly. During assembly, ensure that the ends of the two gel plates are aligned, correctly snap them into the gel frame to secure them, and perform a leak test. Taking a 5 mL system as an example, first add 1 mL of 5% CS solution and 4 mL of 1% copolymer solution to a test tube. Depending on the desired concentration of the PAM/CS/MXene composite conductive hydrogel, add 0 μL, 40 μL, 80 μL, 120 μL, 160 μL, or 200 μL of MXene solution, respectively. Pipette to mix thoroughly and let stand for 15 min. Add 50 μL of 10% APS solution to the test tube, pipette to mix, and then transfer the mixture to a vial or gel casting plate. Place in a 55°C oven for 30 min to obtain six groups of PAM/CS/MXene composite conductive hydrogels with MXene concentrations of 0 μg/mL, 200 μg/mL, 400 μg/mL, 600 μg/mL, 800 μg/mL, and 1000 μg/mL.

### 2.3 SEM observation

After adding the hydrogel solution and mixing thoroughly, the reaction mixture was dispensed into a 24-well plate at a volume of 1 mL per well. The plate was placed in an oven at 55°C for 30 min. Once the hydrogel had fully formed, the gel samples were removed from the plate. The hydrogels were frozen overnight at −20°C. The frozen hydrogels were then placed in a freeze-dryer for 4 h of freeze-drying. The freeze-dried hydrogel samples were mounted onto copper substrates using conductive adhesive, followed by gold sputtering to enhance conductivity.

### 2.4 FTIR detection

A Nicolet IS50 Fourier-transform infrared spectrometer was used to acquire FTIR peak spectra of CS, PAM/CS, and PAM/CS/MXene hydrogels in the range of 500–4000 cm^−1^ at a scanning rate of 2 cm^−1^; the data were then analyzed and calculated using Origin software.

### 2.5 Mechanical property testing

Six sets of PAM/CS/MXene conductive hydrogels with different MXene concentrations were prepared using mold casting. The resulting hydrogel samples were trimmed into cylinders of uniform dimensions (1 cm in diameter and 0.5 cm in thickness), then immersed in ultrapure water overnight to ensure complete hydration and equilibrium. Compression testing was conducted on a universal electronic testing machine using cylindrical specimens (1 cm in diameter and 0.5 cm in thickness), with a compression rate set at 10 mm/min. After zeroing the data, compression mechanical testing was conducted, and the stress–strain response data of the samples were continuously recorded. Statistical analysis and data visualization were performed using software such as Origin and GraphPad Prism. Stress–strain curves were plotted for the six hydrogels of different concentrations, and their corresponding elastic moduli were calculated based on these curves.

### 2.6 Rheological property testing

A HAAKE MARS rotational rheometer equipped with a parallel-plate fixture was used. Under conditions of a fixed frequency of 1 Hz and a plate gap of 1 mm, the test temperature was set to 55°C. Under these conditions, time-sweep oscillatory tests were conducted on six groups of PAM/CS/MXene conductive hydrogels with different MXene concentrations. The intersection points of the time-dependent curves for the storage modulus (G′) and loss modulus (G″) were determined. GraphPad Prism software was used to perform statistical analysis of the experimental data, thereby determining the gelation times for each hydrogel system.

### 2.7 Conductivity testing

Six sets of PAM/CS/MXene composite conductive hydrogel samples with different MXene concentrations were prepared in a 24-well plate. The four-point probe conductivity meter was calibrated using a standard sample. Prior to testing, gently remove any residual moisture from the hydrogel surface. Place the sample on the test platform and use the adjustment mechanism to ensure that the tips of the four probes make uniform and simultaneous contact with the sample surface, then measure the hydrogel's conductivity. Record the conductivity values displayed by the instrument and use GraphPad Prism software to perform statistical analysis and organize the results.

### 2.8 Swelling test

Cut PAM/CS/MXene composite conductive hydrogels with different MXene concentrations into samples of equal volume. Measure the initial mass of the hydrogels using an analytical balance and record it as W0. Immerse the hydrogels in 1×PBS. Select time intervals of 10 min, 2 min, 30 min, 40 min, 50 min, 60 min, and 70 min. At each time point, wipe the PBS from the surface of the hydrogel, weigh the hydrogel again using an analytical balance, and record the mass as W1. Use Microsoft Excel to calculate the swelling ratio (SR) according to the following formula: SR = [(W1 – W0)/W0] × 100%

### 2.9 Cell culture

Remove the cell cryovials from the −80°C freezer and place them in a plastic cup containing 37°C ultrapure water to thaw. Once thawed, transfer them to the cell culture room. When the cells have grown to cover 80–90% of the surface area of a large culture dish, perform the passaging procedure. First, discard the culture medium from the dish and add 2 mL of pre-warmed 1×PBS to wash the cells. After discarding the PBS, add 1 mL of pre-warmed 0.25% trypsin for digestion, limiting the digestion time to 30 s. Add 2 mL of complete medium to stop the digestion reaction. After pipetting gently to mix, transfer the cells to a 5 mL centrifuge tube and centrifuge at 1200 rpm for 5 min. Discard the supernatant, add 1 mL of complete medium to the centrifuge tube to resuspend the cells, and transfer an appropriate amount of the cell suspension to a large culture dish containing 7 mL of complete medium. Mix thoroughly using the cross-stirring method, then place the culture dish in a 37°C incubator for continued culture.

### 2.10 Co-culture of cells and hydrogels

Weigh 3 g of agarose powder, add 4 mL of 50× TAE buffer, and transfer the mixture to a conical flask. Make up the volume to 200 mL with ultrapure water, then autoclave at 121°C for 15 min. Discard the culture medium from the surface of the pre-treated PAM/CS/MXene composite conductive hydrogel. Use a pipette to draw up the sterilized agarose solution and fill the gap between the hydrogel and the well plate. After the agarose solution has solidified, add complete culture medium to the well plate again to keep the hydrogel surface moist. Resuspend the centrifuged cells in 1 mL of complete culture medium, and dilute the cell suspension to the appropriate concentration using complete culture medium as required by the experiment. Discard the complete culture medium from the microplate, and evenly dispense the diluted cell suspension into the wells containing the hydrogel. Mix thoroughly using the cross-stirring method, then place the microplate in a 37°C incubator for cell culture.

### 2.11 Cell viability/death staining

Cultured cells at a density of 2 × 10^5^ cells/mL were co-cultured with PAM/CS/MXene composite conductive hydrogels in a 24-well plate. Once the cells had reached an appropriate growth stage, the culture medium was discarded. 500 μL of pre-warmed 1×PBS was added to each well to wash the cells. Discard the 1×PBS, add 200 μL of Working Solution A to each well to cover the cells, and incubate in a 37°C incubator for 30 min. Discard Working Solution A, add 500 μL of pre-warmed 1×PBS to each well to wash the cells. Add 200 μL of Working Solution B to each well to cover the RSC96 cells, and incubate in a 37°C incubator for 30 min. Discard Solution B, add 500 μL of pre-warmed 1×PBS to each well, and wash the cells. Observe the cells using an LSM900laser confocal microscope and capture images. Process the cell images using software such as ImageJ or GraphPad Prism to count the number of live and dead cells and calculate their ratio.

### 2.12 EdU cell proliferation assay

Cultivate cells at a density of 2 × 10^5^ cells/mL in a 24-well plate together with the PAM/CS/MXene composite conductive hydrogel until the cells reach an appropriate growth stage. Remove Solution A from the EdU Cell Proliferation Assay Kit and prepare a working solution using a 500:1 ratio of complete culture medium to Solution A. Add 200 μL of EdU working solution A to each well, ensuring the cells are covered, and incubate in a 37°C incubator for 2 h. Prepare the DNA staining solution (1× Hoechst 33342 solution) in the dark, using a ratio of 50 parts ultrapure water to 1 part Solution F. Wash the plates and store them at 4°C in the dark. Observe the cells using an LSM900laser confocal microscope and capture images. Process and analyze the cell images using software such as ImageJ or GraphPad Prism.

### 2.13 CCK-8 cell viability assay

Cultivate cells at a density of 2 × 10^5^ cells/mL with the PAM/CS/MXene composite conductive hydrogel in a 24-well plate until the cells reach an appropriate growth stage. Prepare the CCK-8 working solution in the dark, mixing the CCK-8 solution with complete medium at a ratio of 1:9. Incubate both plates in a 37°C incubator for 2 h. Transfer the CCK-8 solution from the 24-well plate to a 96-well plate, adding 100 μL to each well. Place the 96-well plate on a microplate reader pre-warmed to 37°C and measure absorbance at a wavelength of 450 nm. Use software such as ImageJ or Excel to perform statistical analysis of the data.

### 2.14 Cell immunofluorescence staining

Cells were co-cultured with PAM/CS/MXene composite conductive hydrogels in 24-well plates at a density of 2 × 10^5^ cells/mL until reaching the appropriate confluence. Immunostaining blocking solution was added to each well and incubated for 1 h. The staining solution was prepared by diluting Alexa Fluor 488-conjugated phalloidin (green, direct label) and DAPI in primary/secondary antibody dilution buffer at ratios of 1:200 and 1:100, respectively. After removing the blocking solution, 200 μL of staining solution was added to each well and incubated overnight at 4°C. Cells were then observed and imaged using an LSM900 laser scanning confocal microscope. Image processing and statistical analysis were performed using ImageJ, GraphPad Prism, and other software.

### 2.15 Real-time quantitative PCR

Prepare a mixture in a 1:3:5:1 ratio of forward and reverse primers, PCR buffer, SYBR qPCR Master Mix, and cDNA, and place it on ice. On ice, dispense 10 μL of the mixture into each well of the 8-well plate according to the layout. Use forceps to remove the lid from the 8-well plate and replace it. Centrifuge gently for 10 s. Set the program and place the 8-well plate into the real-time quantitative PCR instrument for analysis.

### 2.16 Cell transcriptome sequencing

Cultivate cells at a density of 5 × 10^5^ cells/mL with PAM/CS/MXene composite conductive hydrogels in a 12-well plate for a period of time. Discard the culture medium, add 1 mL of 1×PBS to each well, and wash the cells. Discard the PBS, add 1 mL of Trizol lysis buffer to each well, and gently pipette to ensure complete cell lysis. Transfer the mixture to RNase-free 1.5 mL centrifuge tubes, seal the tube openings with plastic wrap, and store at −80°C. Send the samples to the Suzhou Genewise Genomics Center for transcriptomic sequencing.

### 2.17 Co-culture of DRG neurons with hydrogels

Neonatal Sprague-Dawley (SD) rats (1 day old) were disinfected with 75% ethanol and euthanized by cervical dislocation in a biosafety cabinet. Dorsal root ganglion (DRG) tissue blocks were carefully extracted from the intervertebral foramina using micro-forceps and transferred to a small dish containing dissection solution. Then, 1 mL of type I collagenase (3 mg/mL) was added, and the tissue was gently triturated with a pipette. The dish was subsequently placed in a 37°C cell culture incubator for enzymatic digestion for 30 min, with gentle agitation every 10 min, followed by centrifugation at 1000 rpm for 5 min. The collagenase solution was discarded, and 1 mL of 0.25% trypsin was added for further digestion in the 37°C incubator for 20 min until complete dissociation was achieved. The digestion was terminated by adding 3 mL of complete medium without penicillin-streptomycin (PS). A 200-mesh cell strainer was pre-wetted with PS-free complete medium, through which the cell suspension was filtered, followed by centrifugation at 1000 rpm for 5 min. The resuspended DRG neurons were counted using a hemocytometer and subsequently diluted to a density of 5 × 10^4^ cells/mL.

### 2.18 Fabrication of multichannel nerve guidance conduits

Taking a 5 mL system as an example, 1 mL of 5% chitosan (CS) solution and 4 mL of 1% bisacrylamide mixture were first added to a test tube. Varying amounts of MXene solution were added according to the desired MXene concentrations. The mixture was pipetted to homogeneity and allowed to stand for 15 min. Subsequently, 50 μL of 10% ammonium persulfate (APS) solution was added, and after thorough mixing, the solution was transferred into a multichannel mold. The mold was placed in a 55°C oven for 30 min to allow polymerization. Upon demolding, PAM/CS/MXene composite conductive hydrogel nerve guidance conduits were obtained.

### 2.19 Construction of rat sciatic nerve defect bridging model

Twenty Sprague-Dawley (SD) rats were randomly divided into four groups (n = 5 per group): defect group (Blank), 0 μg/mL MXene group, 600 μg/mL MXene group, and autograft group (Autograft). The left hind limb was designated as the experimental side. The rats were anesthetized by intraperitoneal injection of compound anesthetic. After anesthesia, the surgical hind limb was shaved to expose the skin. The skin was disinfected with iodophor cotton balls, followed by wiping with 75% ethanol for deiodination. An oblique incision of approximately 3 cm was made to expose the surgical area. The fascia and muscle gaps were bluntly dissected to locate the sciatic nerve. After measuring the nerve length with a ruler, the sciatic nerve was transected and a 10 mm segment was excised to create a nerve defect. In the Blank group, the nerve was transected and bridged with a silicone tube only. In the 0 μg/mL and 600 μg/mL MXene groups, the corresponding hydrogel nerve guidance conduits were inserted into the silicone tubes for bridging. In the Autograft group, a 10 mm nerve segment was reversed and used for bridging. During this process, 8-0 sutures were used for anastomosis. The muscle and skin layers were closed layer by layer with 4-0 surgical sutures. After recovery from anesthesia, the rats were housed individually and monitored regularly for health status and wound healing.

### 2.20 Behavioral testing

At three time points—4, 8, 12 weeks post-surgery—rats were placed at the entrance of the gait analysis system and allowed to walk voluntarily along the track to the endpoint. Simultaneously, the Cat Walk software was used to capture and record the walking trajectories of each group of ras in real time. The acquired data were analyzed using software such as Excel and GraphPad Prism to calculate the Sciatic Nerve Functional Index (SFI) for each group of rats at different time points post-surgery.

$$\begin{array}{c} \text{SFI}=-38.3\times \frac{PL_{E}‐PL_{N}}{PL_{N}}+109.5\times \frac{TS_{E}‐TS_{N}}{TS_{N}}+13.3\times \frac{\text{IT}S_{E}‐\text{IT}S_{N}}{\text{IT}S_{N}}-8.8\; \end{array}$$

Measurement parameters: length from heel to toe (Paw Length, PL), distance between the 1st and 5th toes (Toe Spread, TS), and distance between the 2nd and 4th toes (Intermediate Toe Spread, ITS).

### 2.21 CMAP measurement

Twelve weeks post-surgery, rats were anesthetized via intraperitoneal injection, and the hair on the surgical side and the contralateral side was shaved using an electric shaver. The bridged segment of the sciatic nerve and the contralateral normal sciatic nerve were exposed.

A bipolar electrode was placed approximately 3 mm proximal to the proximal anastomosis site of the nerve-tube graft and used as the stimulation electrode. A coaxial needle electrode was used as the recording electrode, inserted into the midpoint of the thickest part of the gastrocnemius muscle belly and the corresponding tendon, respectively, to a depth of approximately 5 mm. The ground electrode was clamped to the rat's skin. Set the pulse width of the conductive to 0.1 ms and the current intensity to 5.0 mA, and manually trigger the delivery of a single stimulus. Record and observe the CMAP waveform of the gastrocnemius muscle. Use software such as Excel or GraphPad Prism to statistically analyze the peak amplitude and latency.

### 2.22 Determination of wet weight ratio

At 12 weeks post-surgery, following completion of electrophysiological testing, rats were euthanized via intraperitoneal injection of an overdose of a combination anesthetic. After depilation, the skin of the hind limbs was incised to expose the gastrocnemius muscle. The muscle was bluntly dissected, and the gastrocnemius muscles from the operated and contralateral sides were removed, with excess connective tissue excised. The wet weights were measured using an electronic balance and recorded. Statistical analysis and calculation of the wet weight ratio of the target muscle were performed using software such as Excel and GraphPad Prism.

“Wet weight ratio = Wet weight of the operated side / Wet weight of the contralateral side × 100%”

### 2.23 H&E staining of the gastrocnemius muscle

After weighing, the gastrocnemius muscle was immersed in 4% paraformaldehyde and fixed overnight in a 4°C refrigerator to ensure thorough fixation of the tissue. Gradual dehydration was performed using 5%, 15%, and 30% sucrose solutions, with a dehydration time of 24 h. The gastrocnemius muscle was embedded in OCT and sectioned into 10-μm-thick frozen sections. The sections were mounted on adhesive slides and dried overnight in a 37°C oven, then stored at −20°C. Place the sections in a hematoxylin solution for 8 min to stain the cell nuclei, then rinse five times with distilled water for 1 min each. Discard the staining solution and place the sections in a 1% hydrochloric acid-ethanol solution for 10 s to decolorize. Rinse with distilled water for 15 min to halt denaturation and perform decolorization. Place the sections in an eosin stain solution for 40 s to stain the cytoplasm red. For tissue dehydration and clearing, rinse sequentially with 75%, 85%, 95%, and 100% ethanol solutions, twice each for 5 min. Rinse twice with xylene, 5 min each. Apply a drop of neutral resin to the section, cover with a coverslip to seal, and dry overnight in a fume hood for storage. Subsequent imaging was performed using a Zeiss microscope, and data were analyzed using software such as ImageJ and GraphPad Prism.

### 2.24 Transparization and staining of regenerated nerve tissue

The sciatic nerves fixed in 4% paraformaldehyde were sent to Yifu Biotechnology Nantong Co., Ltd. for tissue transparization and staining. The sciatic nerves were immersed in 25% Quadrol and decolorized on a shaking incubator at 37°C for 2 days, then rinsed with 1×PBS. The sciatic nerve was immersed in Delipidation Buffer for tissue clearing and incubated on a shaking incubator at 45°C for 7 days, followed by rinsing with 1×PBS. The sample was immersed in Antibody Blocking Solution containing 5% donkey serum (from the same species as the secondary antibody) and blocked by shaking at 37°C for 2 days. Prepare the antibody diluent. Following the dilution ratios for antibodies used in transverse section immunohistochemical staining of the sciatic nerve, prepare the primary and secondary antibody staining solutions using the diluent. Incubate the sciatic nerve with the primary antibody at 37°C for 6 days, and with the secondary antibody at 37°C for 6 days. First, transfer the sample to 20 mL of 50% EasyIndex, add DAPI, and incubate on a shaking incubator at 37°C for 1 day; then transfer to 20 mL of 100% EasyIndex and incubate on a shaking incubator at 37°C for another 1 day until completely transparent. Embed in 1% agarose gel for imaging; soak the embedded agarose block in 100% EasyIndex overnight before photographing and observing.

### 2.25 TEM observation of the ultrastructure of regenerating axons

At 12 weeks post-surgery, rats were euthanized via intraperitoneal injection of an overdose of a combination anesthetic. The sciatic nerve was pre-fixed in 4% glutaraldehyde solution for 2 h, then rinsed six times in 0.1 M sodium diarsenate buffer, 15 min per rinse. The specimens were post-fixed in 1.0% osmic acid for 2 h, followed by six rinses with distilled water, each lasting 5 min. Gradient dehydration was performed using 30%, 50%, 70%, 80%, 95%, and 100% ethanol solutions, with two passes in each concentration, each lasting 15 min. Dehydrate with propylene oxide twice, 30 min each. Prepare a solution with a 1:2 ratio of EPON 812 to propylene oxide and infiltrate the sciatic nerve for 3 h. Prepare a solution with a 2:1 ratio of EPON 812 to propylene oxide and infiltrate the sciatic nerve for 5 h. Infiltrate the sciatic nerve with the embedding medium overnight. Sections were prepared using the semi-thin sectioning method (section thickness: 2 μm) for localization, followed by ultrathin sectioning (section thickness: 70–90 nm). Double staining was performed using 4% uranyl acetate and 0.3% lead citrate. The ultrastructure of regenerating nerves was observed using an HT7700 transmission electron microscope. Statistical analysis was performed using software such as ImageJ and GraphPad Prism.

### 2.26 Statistical analysis

In this study, statistical analyses were performed using three or more parallel groups to minimize error and ensure the reliability of the experimental results. GraphPad Prism was used for statistical analysis. Experimental data are presented as mean ± standard error of the mean (mean ± SEM). Differences between groups were analyzed using one-way analysis of variance (One-Way ANOVA). *P < 0.05, **P < 0.01, ***P < 0.001, ****P < 0.0001.

## 3 Results

### 3.1 Fabrication and characterization of PAAm/CS/MXene hydrogels

PAAm/CS and PAAm/CS/MXene hydrogels were fabricated via free-radical polymerization (Fig. 1A). As MXene concentration increased from 0 to 1000 μg/mL, the hydrogels progressively darkened in color while retaining excellent injectability (Fig. 1B). Transmission electron microscopy (TEM) confirmed the typical 2D layered nanosheet morphology of MXene (Fig. 1C). Fourier transform infrared (FTIR) spectroscopy verified successful integration of MXene into the hydrogel network, with characteristic peaks consistent with intermolecular interactions between MXene and the PAAm/CS matrix (Fig. 1D). Mechanical characterization revealed that Young's modulus rose significantly with increasing MXene loading (Fig. 1E, ****P < 0.0001). Stress-strain curves further showed enhanced tensile strength and ductility in MXene-doped hydrogels compared to the PAAm/CS control (Fig. 1F), indicating that MXene nanosheets act as effective reinforcing fillers to improve the mechanical robustness of the hydrogel network. Swelling ratio measurements demonstrated that all hydrogel formulations rapidly absorbed water and reached equilibrium within ∼60 min (Fig. 1G). Higher MXene concentrations slightly reduced the equilibrium swelling ratio relative to the 0 μg/mL control, suggesting that MXene incorporation modulates the water uptake capacity of the hydrogel by densifying the polymer network. Electrical conductivity increased monotonically with MXene concentration (Fig. 1H), confirming that MXene serves as a conductive nanofiller within the insulating PAAm/CS matrix.

**Fig. 1:**
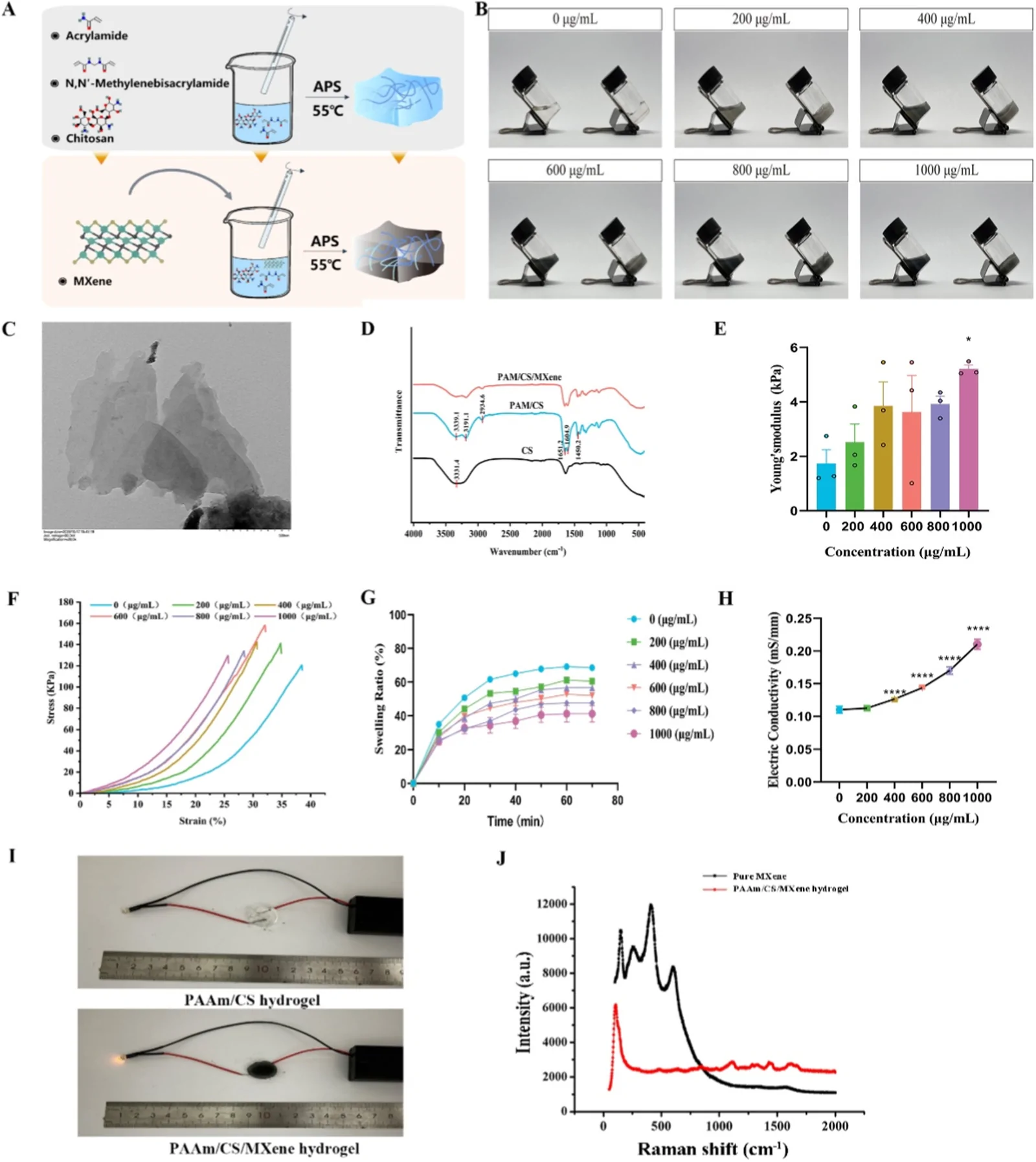
Preparation and characterization of PAAm/CS/MXene composite hydrogels. (A) Schematic illustration of the preparation process of PAAm/CS and PAAm/CS/MXene hydrogels via free-radical polymerization. (B) Digital photographs of hydrogels with different MXene concentrations (0–1000 μg/mL). (C) TEM image of MXene nanosheets. (D) FTIR spectra of CS, PAAm/CS, and PAAm/CS/MXene hydrogels. (E) Young's modulus and (F) stress-strain curves of hydrogels with varying MXene concentrations. (G) Swelling ratio of hydrogels over time. (H) Electrical conductivity of hydrogels as a function of MXene concentration. I) Photographs demonstrating the conductivity of PAAm/CS and PAAm/CS/MXene (600 μg/mL) hydrogels in a circuit with an LED indicator. (Data are presented as mean ± SD; ****P < 0.0001. (J) Raman spectra of pure MXene and PAAm/CS/MXene hydrogel.

Raman spectroscopy was used to characterize the structures of pristine MXene nanosheets and PAM/CS/MXene composite hydrogel, to evaluate whether MXene was oxidized during hydrogel fabrication. Functional testing showed that the PAAm/CS/MXene hydrogel could power an LED light, whereas the non-conductive PAAm/CS control could not (Fig. 1I), demonstrating the potential of these composite hydrogels for flexible electronic and bioelectronic applications. As shown in Fig. 1J, Raman results confirmed successful integration of MXene nanosheets into the PAAm/CS hydrogel, and no obvious MXene oxidation was observed during hydrogel preparation.

Rheological time-sweep measurements revealed that both the storage modulus (G′, black dashed line) and loss modulus (G″, red dashed line) increased rapidly after polymerization initiation, confirming successful gel formation across all MXene concentrations (Fig. 2A). Notably, higher MXene loadings correlated with faster gelation kinetics and higher final G′ values, indicating that MXene reinforces the hydrogel network and accelerates crosslinking. In all formulations, G′ consistently exceeded G″, confirming the elastic, solid-like nature of the hydrogels. Scanning electron microscopy (SEM) images demonstrated that MXene doping significantly modified the hydrogel's porous microstructure (Fig. 2B). As MXene concentration increased from 0 to 1000 μg/mL, the average pore area decreased progressively (from 1069 μm^2^ to 72 μm^2^), accompanied by a denser, more compact pore network. Quantitative pore area analysis confirmed a gradual reduction in pore size with increasing MXene loading, reflecting the nanofiller-induced densification of the polymer matrix. The pore-size data from SEM cross-section images are used for relative comparison among groups rather than as absolute values, as discussed below.

**Fig. 2:**
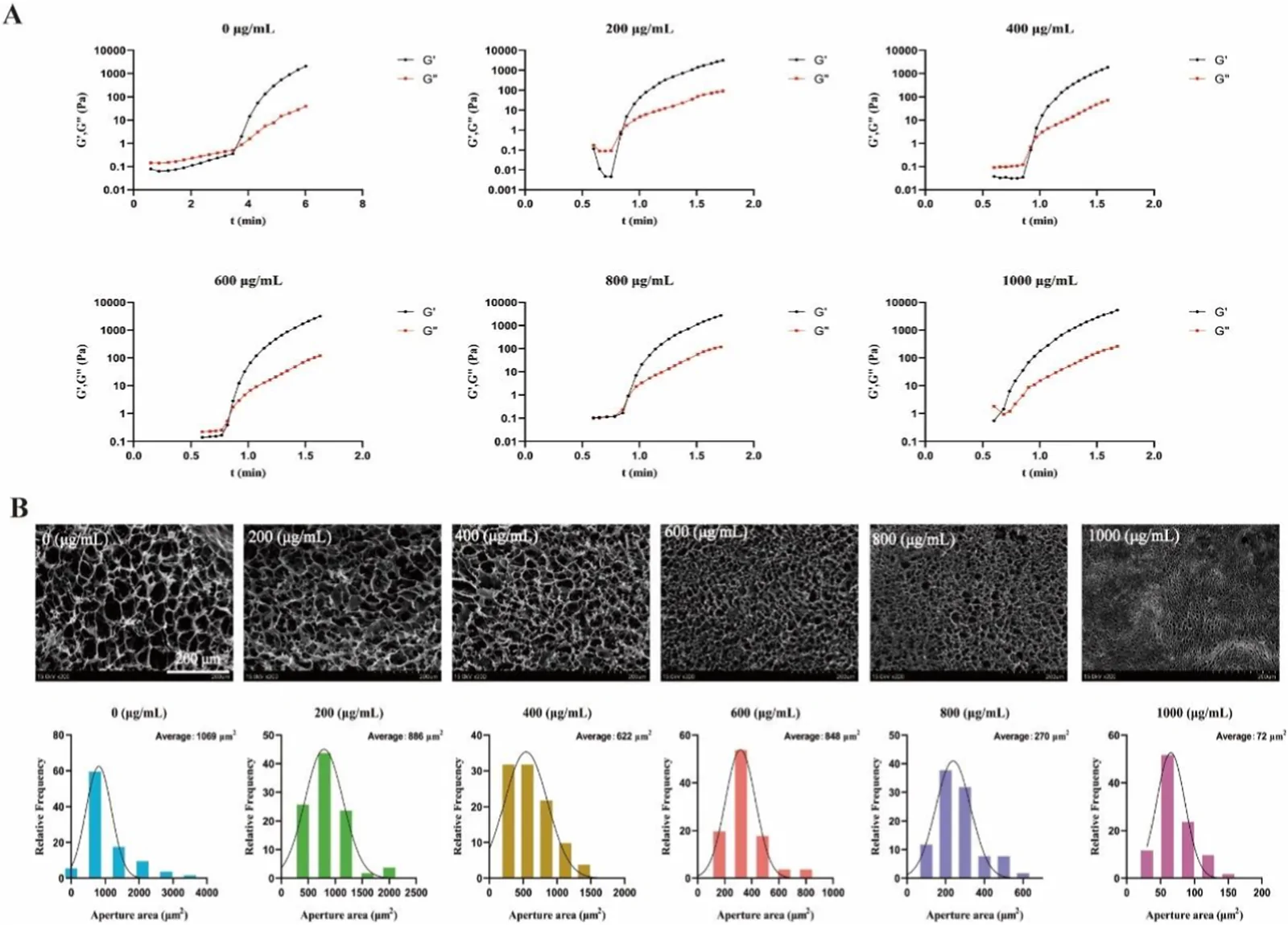
(A) Time sweep rheology showing G′ (black) and G″ (red) at different concentrations. Gelation time decreases with increasing concentration. (B) SEM images and pore size distributions of hydrogels formed at 0–1000 μg/mL. Higher concentrations produce denser networks with smaller pores (scale bar: 200 μm). Note: Pore area statistics from SEM cross-section images are used only for relative trend comparison among groups, not for absolute pore-size quantification. Sample dehydration during freeze-drying may induce partial pore collapse of the hydrogel.

### 3.2 Cytocompatibility of PAAm/CS/MXene hydrogels with neural cells

Next, we evaluated the cytocompatibility of PAAm/CS/MXene hydrogels with two neural cell lines, RSC96 and PC12, across a range of MXene concentrations (0–1000 μg/mL) and time points (12, 24, and 48 h). Cell viability was first assessed by CCK-8 assay (Fig. 3A and B). In RSC96 cells, the optical density (OD) values at 450 nm increased over time, with no significant reduction observed at any MXene concentration up to 1000 μg/mL, indicating robust cell proliferation (Fig. 3A). Similarly, PC12 cells maintained high viability and proliferative activity across all MXene concentrations and time points, confirming the non-cytotoxic nature of the hydrogel formulations (Fig. 3B).

**Fig. 3:**
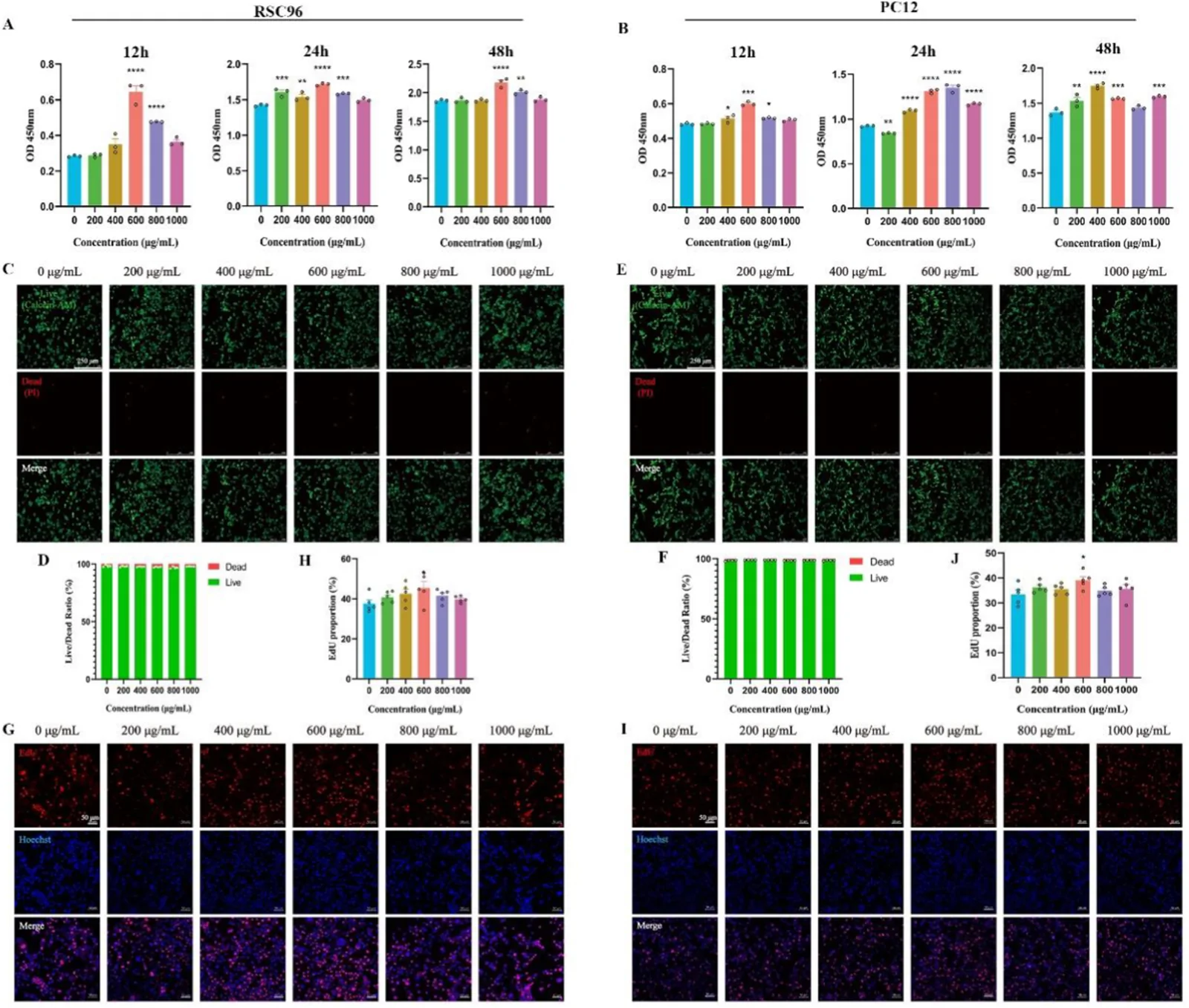
Biocompatibility and proliferation of RSC96 and PC12 cells on hydrogels at different concentrations. (A, B) CCK-8 assay showing cell viability at 12, 24, and 48 h. (C, E) Live/Dead staining (Calcein-AM/PI) of RSC96 and PC12 cells, respectively. (D, F) Quantification of live/dead cell ratios. (G, I) EdU staining for cell proliferation. (H, J) Quantification of EdU-positive cell proportions. Scale bars: 250 μm (C, E); 50 μm (G, I). Data are presented as mean ± SD. *P < 0.05, **P < 0.01, ***P < 0.001,****P < 0.0001.

Live/dead staining further validated these findings (Fig. 3C–F). For both cell lines, the vast majority of cells were labeled as live (green), with only sparse dead cells (red) detected even at the highest MXene concentration (1000 μg/mL). Quantitative analysis confirmed that the live/dead ratio remained above 95% for all conditions (Fig. 3D–F), demonstrating excellent cytocompatibility. EdU incorporation assays were performed to assess cell proliferation (Fig. 3G–J). Consistent with the CCK-8 results, the proportion of EdU-positive (proliferating) cells remained high across all MXene concentrations, with only a slight, non-significant decrease observed at 1000 μg/mL relative to the 0 μg/mL control (Fig. 3H–J). Collectively, these results confirm that PAAm/CS/MXene hydrogels exhibit outstanding cytocompatibility and support the proliferation of neural cells.

### 3.3 MXene doping promotes RSC96 Schwann cell morphological elongation and differentiation

To further characterize the effects of PAAm/CS/MXene hydrogels on neural cell morphology and differentiation, we performed phalloidin staining and gene expression analysis in RSC96 Schwann cells. Phalloidin staining revealed that RSC96 cells maintained healthy, spread morphology across all MXene concentrations (0–1000 μg/mL) and time points (12, 24, and 48 h) (Fig. 4A). Quantitative morphometric analysis showed that cell aspect ratio, spreading area, and circularity were dynamically modulated by MXene loading (Fig. 4B–D). At 12 h, cells at 600 μg/mL MXene exhibited the highest aspect ratio and spreading area, while circularity increased with higher MXene concentrations, indicating enhanced cell polarization and elongation. Similar trends were observed at 24 and 48 h, confirming that MXene doping promotes a more elongated, pro-regenerative cell morphology conducive to neural repair. We next evaluated the expression of key Schwann cell differentiation and myelination-related genes (MBP, NGF, PMP22) at 12 h (Fig. 4E). Quantitative RT-PCR analysis demonstrated that MXene supplementation significantly upregulated the expression of MBP, NGF, and PMP22, with peak expression observed at 600 μg/mL MXene. These findings indicate that PAAm/CS/MXene hydrogels not only support cell viability but also actively promote Schwann cell differentiation and the expression of neurotrophic factors.

**Fig. 4:**
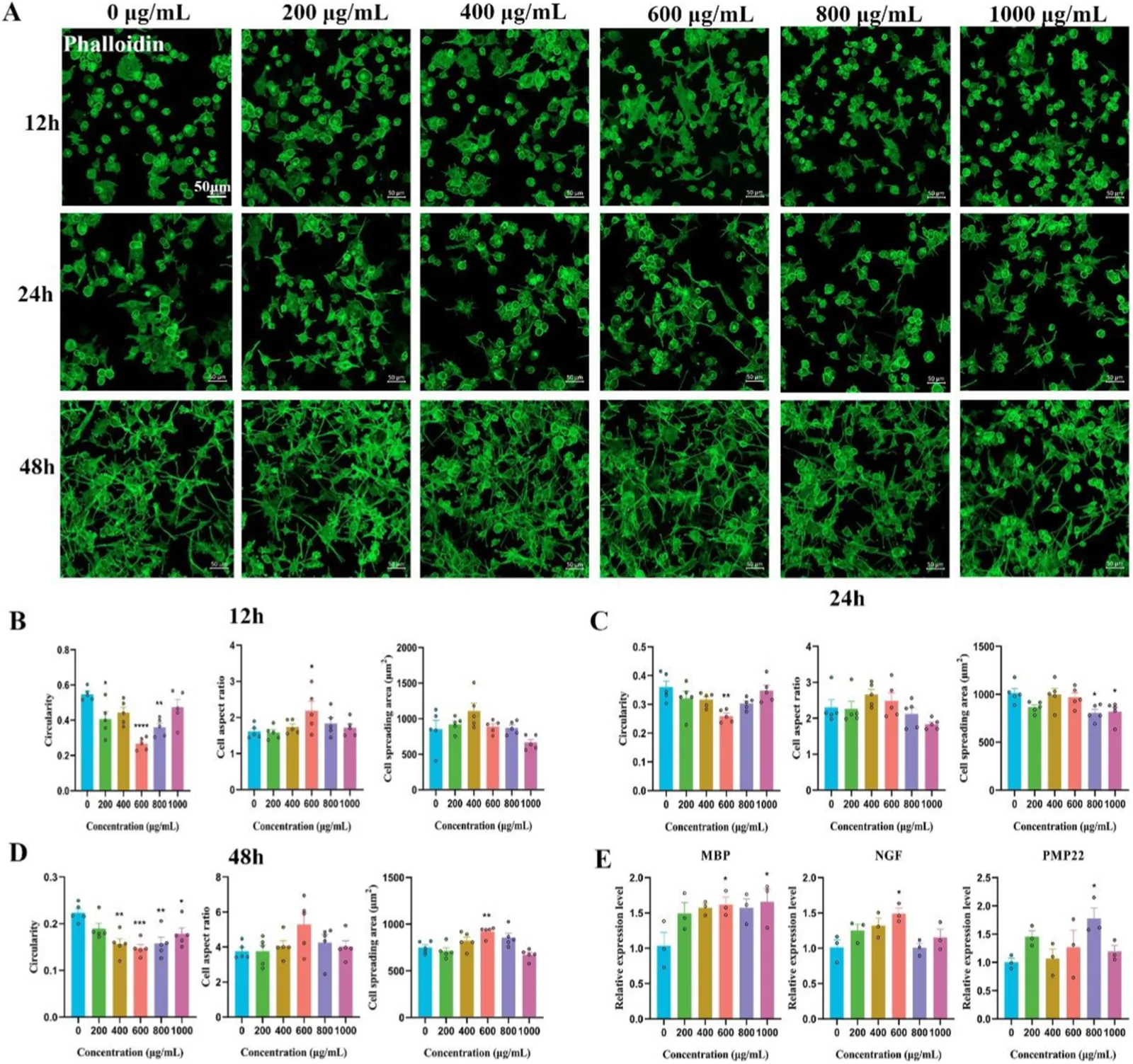
Morphological analysis and gene expression of RSC96 cells on hydrogels at different concentrations. (A) Phalloidin staining of cell cytoskeleton at 12, 24, and 48 h (scale bar: 50 μm). (B–D) Quantification of cell circularity, aspect ratio, and spreading area at 12, 24, and 48 h, respectively. (E) Relative mRNA expression levels of MBP, NGF, and PMP22. Data are presented as mean ± SD. *P < 0.05, **P < 0.01, ***P < 0.001,****P < 0.0001.

### 3.4 Optimal MXene concentration promotes neurite outgrowth in PC12 neuronal cells

Building on the observed effects of MXene doping on Schwann cell morphology and differentiation, we next assessed neurite outgrowth and neuronal gene expression in PC12 cells cultured on PAAm/CS/MXene hydrogels. Phalloidin staining revealed extensive neurite formation across all MXene concentrations (0–1000 μg/mL) and time points (12, 24, and 48 h) (Fig. 5A). Quantitative analysis demonstrated that both mean neurite length and longest neurite length were significantly increased at 600 μg/mL MXene compared to the 0 μg/mL control at all time points (Fig. 5B–D). Peak neurite outgrowth was observed at 600 μg/mL, with further increases in MXene concentration (800–1000 μg/mL) leading to a gradual reduction in neurite length, suggesting an optimal concentration for promoting neurite extension. Consistent with the morphological findings, gene expression analysis of GAP43, a key marker of neurite outgrowth and neuronal development, showed significant upregulation at 600 μg/mL MXene (Fig. 5E). These results indicate that PAAm/CS/MXene hydrogels, particularly at 600 μg/mL MXene, effectively promote neurite outgrowth and neuronal differentiation.

**Fig. 5:**
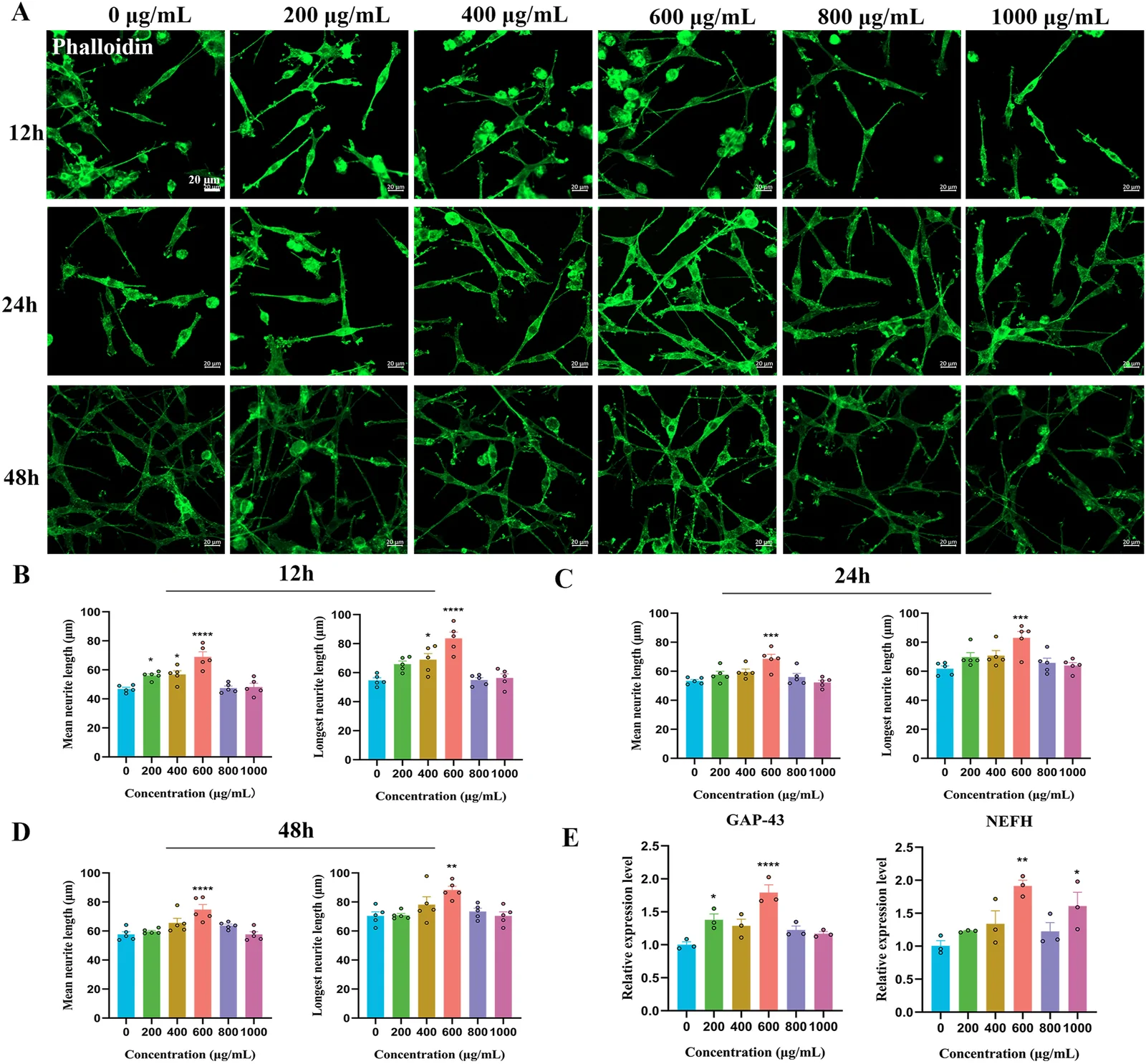
Neurite outgrowth and neuronal differentiation of PC12 cells on hydrogels at different concentrations. (A) Phalloidin staining of cell morphology at 12, 24, and 48 h (scale bar: 20 μm). (B–D) Quantification of mean and longest neurite lengths at 12, 24, and 48 h, respectively. (E) Relative mRNA expression levels of GAP-43 and NEFH. Data are presented as mean ± SD. *P < 0.05, **P < 0.01, ***P < 0.001, ****P < 0.0001.

### 3.5 PAAm/CS/MXene hydrogels enhance axonal outgrowth in primary dorsal root ganglion neurons

To validate the pro-neurite outgrowth effects of PAAm/CS/MXene hydrogels in a more physiologically relevant model, we cultured primary dorsal root ganglion (DRG) neurons on hydrogels with varying MXene concentrations (0–1000 μg/mL) and performed immunofluorescent staining for neurofilament 200 (NF200), a marker of mature neuronal axons. Immunofluorescent imaging revealed extensive neurite network formation across all groups, with the most robust axonal outgrowth observed at 600 μg/mL MXene (Fig. 6A). Quantitative analysis confirmed that both mean neurite length and longest neurite length were significantly increased at 600 μg/mL compared to the 0 μg/mL control (Fig. 6B and C), consistent with our previous findings in PC12 cells. Higher MXene concentrations (800–1000 μg/mL) resulted in reduced neurite length, further supporting the notion of an optimal MXene loading (600 μg/mL) for promoting neuronal regeneration.

**Fig. 6:**
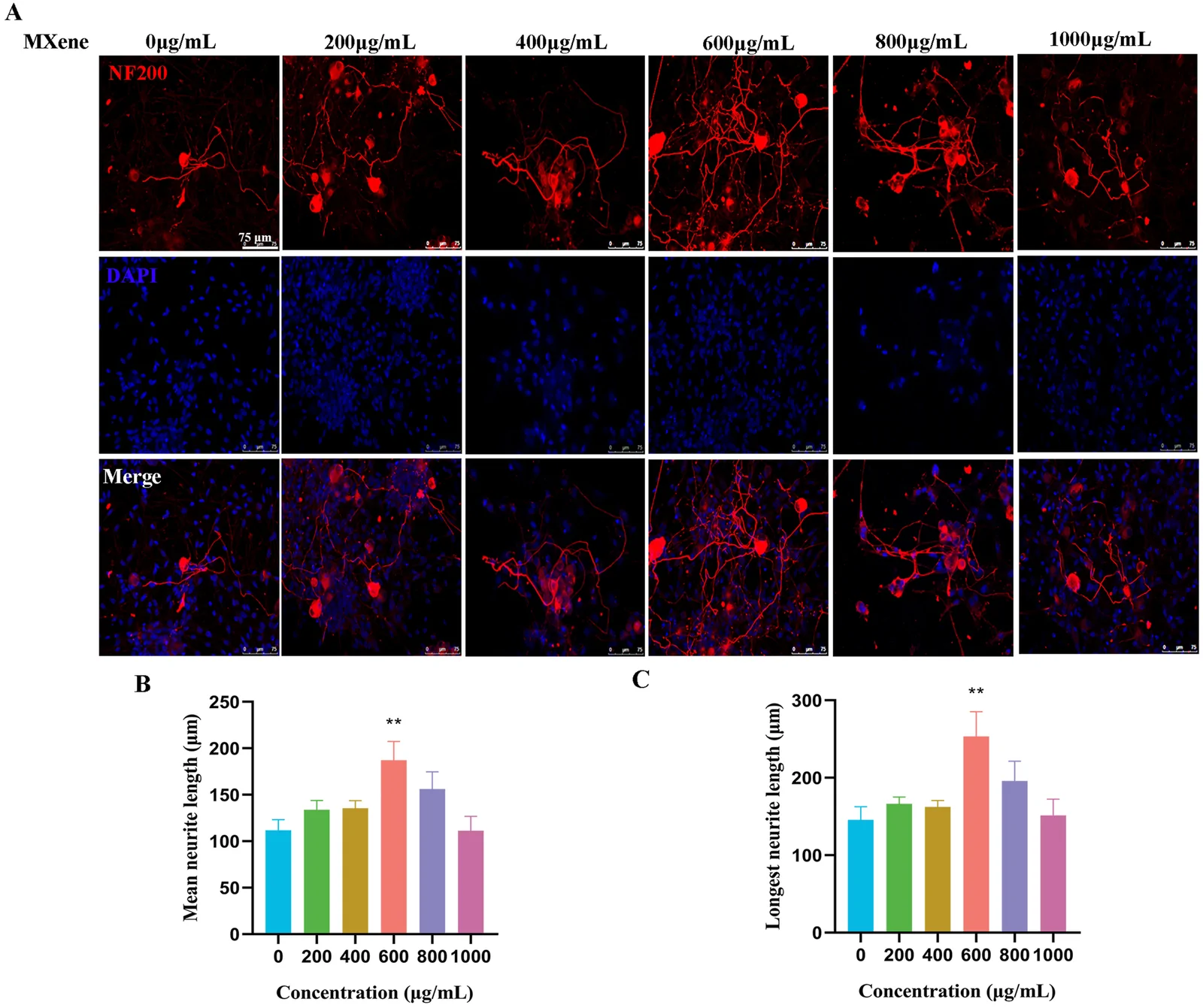
Neurite outgrowth of DRG neurons on MXene-containing hydrogels at different concentrations. (A) Immunofluorescence staining of NF200 (red) and DAPI (blue) at 7 days (scale bar: 75 μm). (B, C) Quantification of mean and longest neurite lengths, respectively. Data are presented as mean ± SD. **P < 0.01.

### 3.6 Transcriptome analysis reveals MXene-induced activation of integrin-mediated signaling pathways in RSC96 and PC12 cells

To elucidate the molecular mechanisms underlying the enhanced cellular responses to the optimized MXene hydrogel, transcriptome sequencing was performed on RSC96 Schwann cells and PC12 neurons cultured on 600 μg/mL MXene hydrogel versus control (0 μg/mL). Differential expression analysis revealed substantial transcriptomic reprogramming in both cell types. In RSC96 cells, 3549 significant DEGs were identified, with 1955 upregulated and 1594 downregulated (Fig. 7A). PC12 cells showed 2025 significant DEGs, including 913 upregulated and 1112 downregulated genes (Fig. 7B). Hierarchical clustering demonstrated clear separation between experimental and control groups, indicating robust and consistent transcriptional changes induced by the MXene hydrogel. GO enrichment analysis of RSC96 cells revealed significant enrichment in intracellular signal transduction, MAPK cascade, cell-matrix adhesion, and axon guidance, with cellular components localized to neuron projection, synapse, focal adhesion, and integrin complex (Fig. 7C). Molecular functions highlighted kinase activity, actin binding, and PI3K regulator activity, while KEGG analysis confirmed enrichment of ECM-receptor interaction, focal adhesion, PI3K-Akt, and MAPK signaling pathways (Fig. 7E). Similarly, PC12 cells showed enrichment in nervous system development, cell adhesion, and axon guidance, with components including cell projection, cytoskeleton, and focal adhesion, and activation of PI3K-Akt, calcium signaling, and axon guidance pathways (Fig. 7D–F). Notably, both cell types converged on integrin-mediated adhesion, PI3K-Akt signaling, and cytoskeletal reorganization, suggesting that these cascades may represent potential key signaling events underlying the pro-regenerative cellular behaviors triggered by the conductive MXene hydrogel. qPCR validation confirmed significant upregulation of *Akt1*, *Integrin β1*, *Ptk2* (FAK), and *Sema3a* in both RSC96 and PC12 cells on the 600 μg/mL hydrogel (Fig. 7E and F). These transcriptomic and qPCR results indicate that the optimized MXene hydrogel is associated with transcriptional upregulation of the integrin-mediated PI3K-Akt signaling and focal adhesion kinase (FAK/Ptk2) pathways, as well as elevated expression of semaphorin 3A (Sema3a), a critical axon guidance molecule. These findings suggest a potential mechanistic association between MXene-modulated cellular transcriptional profiles and improved Schwann cell function, neuronal differentiation, and directed axonal growth. However, further functional intervention experiments are required to verify the causal roles of these signaling cascades.

**Fig. 7:**
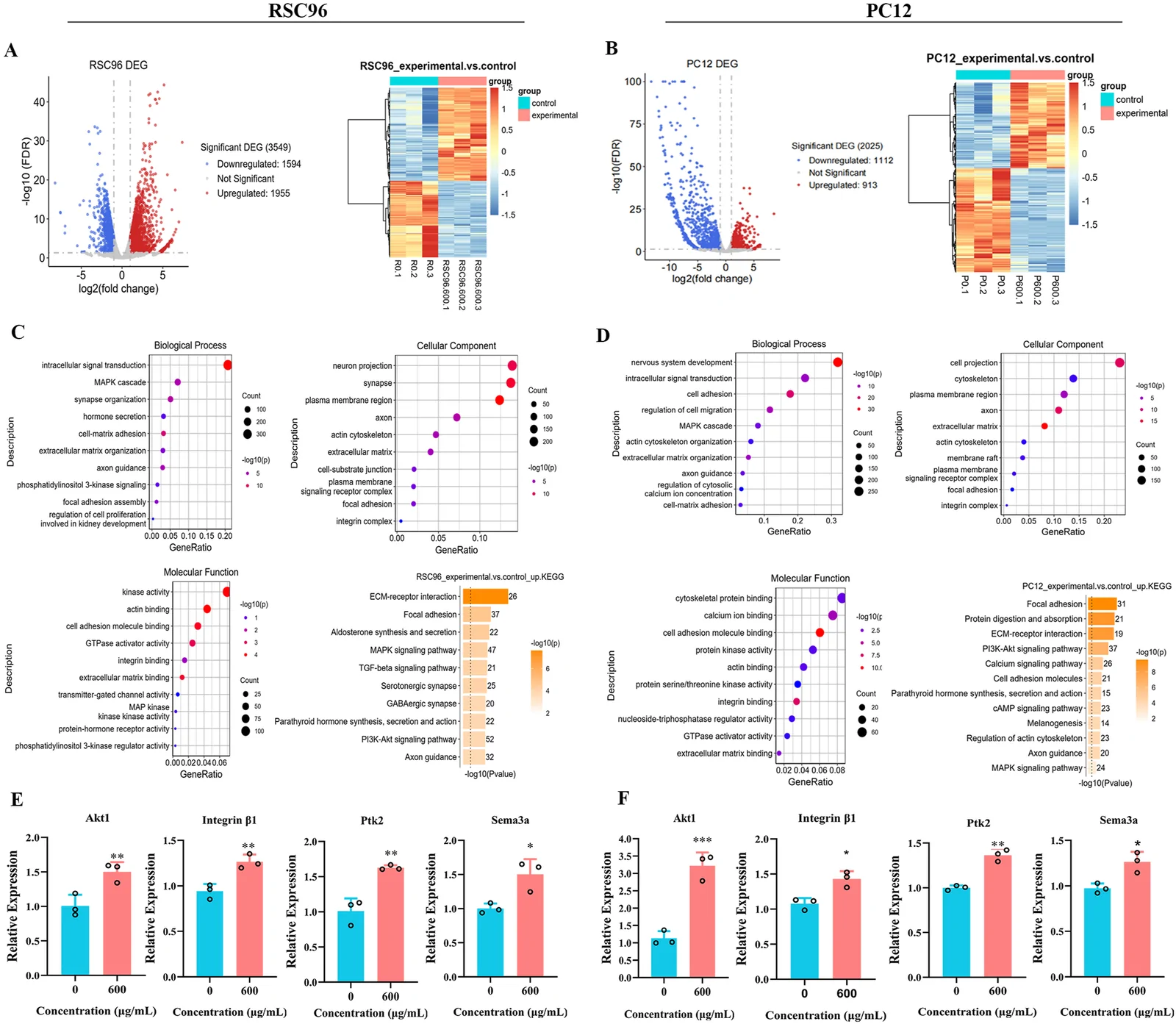
Transcriptome analysis of RSC96 and PC12 cells on the optimized MXene hydrogel. (A, B) Volcano plots and heatmaps showing differentially expressed genes (DEGs) in RSC96 and PC12 cells (600 μg/mL vs. 0 μg/mL). (C–F) GO enrichment analysis of biological process, cellular component, and molecular function, together with KEGG pathway analysis for RSC96 (C, E) and PC12 (D, F) cells. (G, H) GSEA plots validating enrichment of PI3K-Akt, ECM-receptor interaction, and calcium signaling pathways. (I, J) qPCR validation of *Akt1*, *Integrin β1*, *Ptk2*, and *Sema3a* expression in RSC96 and PC12 cells, respectively. Data are presented as mean ± SD. *P < 0.05, **P < 0.01, ***P < 0.001.

### 3.7 PAAm/CS/MXene hydrogel nerve conduit promotes motor functional recovery and tissue regeneration in rat sciatic nerve defect

The macroscopic mechanical performance of the multi-channel PAAm/CS/MXene hydrogel conduit was further assessed. As shown in Fig. S1, the tubular hydrogel conduit can sustain bending and pinching deformations, and fully restore its original shape after load release. Such favorable flexibility and shape-recovering capability are beneficial for surgical manipulation and allow the conduit to adapt to the dynamic soft-tissue microenvironment after implantation. To evaluate the in vivo regenerative efficacy of the hydrogel conduits, a 10 mm rat sciatic nerve defect model was established (Fig. 8A). The conduits exhibited favorable handling properties and porous microstructures, supporting stable bridging of the nerve gap. Over the 12-week follow-up, the sciatic functional index (SFI) in the 600 μg/mL group progressively improved, with significantly higher values than the 0 μg/mL and Blank groups at all time points (Fig. 8B), and gradually approaching the level of the autograft group. Pairwise statistical comparisons were performed among the 0 μg/mL MXene, 600 μg/mL MXene, and autograft groups for all functional and electrophysiological endpoints. Consistent with the SFI results, footprint analysis revealed better walking coordination in the 600 μg/mL group (Fig. 8C). Electrophysiological tests showed that the 600 μg/mL group exhibited significantly increased CMAP amplitude and reduced latency compared with the 0 μg/mL and Blank groups (Fig. 8D–E), indicating enhanced motor nerve conduction function. At 12 weeks, the gastrocnemius muscles in the 600 μg/mL group showed less atrophy and significantly higher relative wet weight than the 0 μg/mL and Blank groups (Fig. 8F–G), demonstrating effective prevention of denervation-induced motor target muscle atrophy. Collectively, these results confirm that the PAAm/CS/MXene (600 μg/mL) hydrogel conduits significantly promote motor functional recovery and motor axon-mediated tissue regeneration in vivo, with regenerative indicators showing no significant statistical difference from those of the autograft group. Notably, SFI, CMAP, and gastrocnemius muscle assessments primarily reflect motor nerve recovery and cannot characterize sensory axon regeneration, sensory target reinnervation, or tactile/nociceptive functional restoration.

**Fig. 8:**
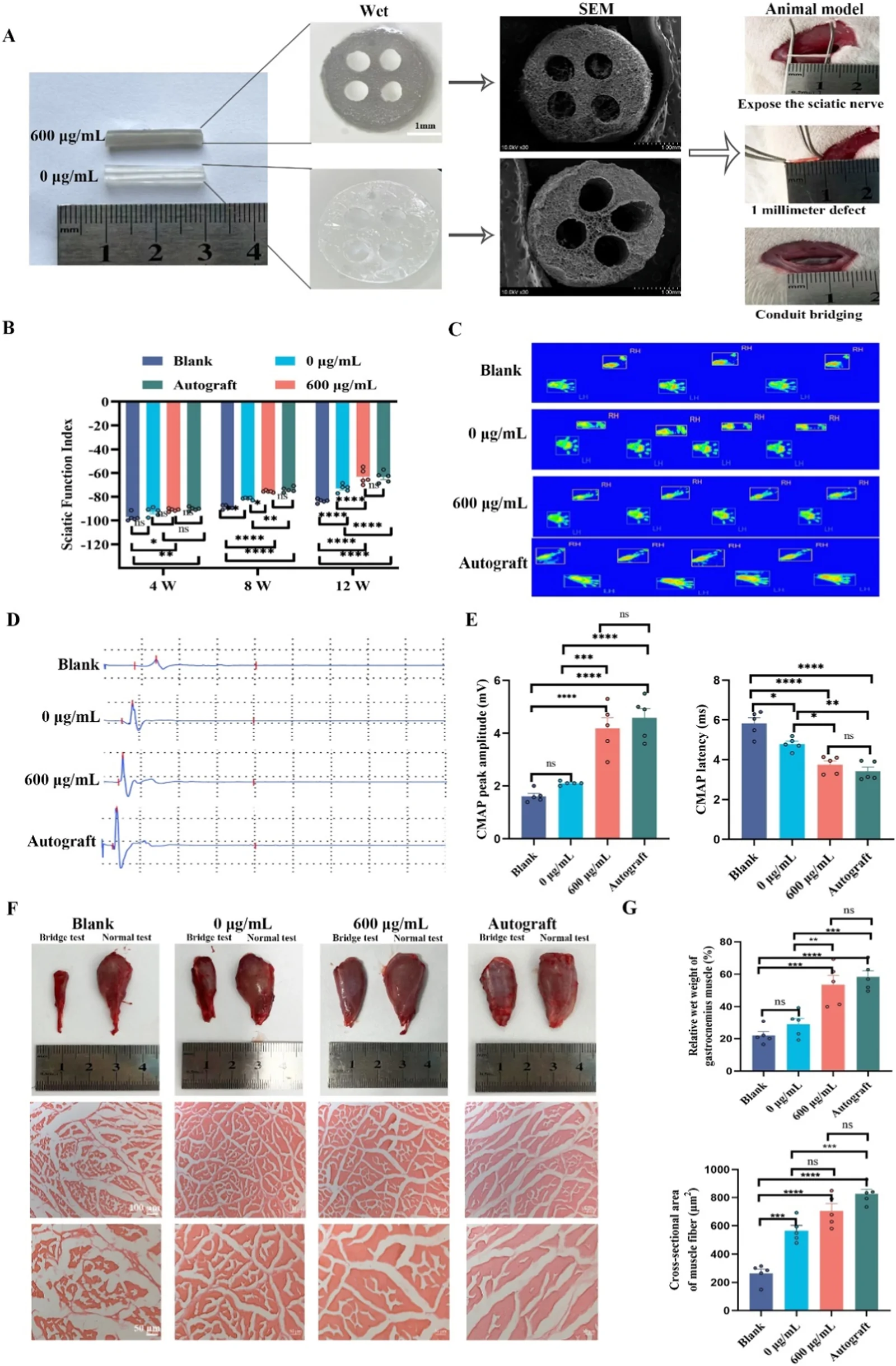
In vivo functional and regenerative performance of the PAAm/CS/MXene hydrogel conduits in a rat sciatic nerve defect model. (A)Schematic of conduit fabrication, morphology (wet state), SEM microstructure, and implantation procedure for a 10 mm rat sciatic nerve defect model. (B) Sciatic functional index (SFI) of rats in the Blank, 0 μg/mL, 600 μg/mL, and autograft groups at 4, 8 and 12 weeks post-implantation (n = 5, *P < 0.05, **P < 0.01, ****P < 0.0001). (C) Representative footprint images of each group at 12 weeks. (D) Representative compound muscle action potential (CMAP) waveforms. (E) Quantitative analysis of CMAP peak amplitude and latency (n = 5, **P < 0.01, ****P < 0.0001). (F) Gross morphology of the gastrocnemius muscles in each group at 12 weeks. (G) Quantitative analysis of relative wet weight of the gastrocnemius muscle (n = 6, **P < 0.01, ***P < 0.001, ****P < 0.0001).

### 3.8 PAAm/CS/MXene hydrogel nerve conduit promotes in vivo regeneration of myelinated nerve fibers

To further investigate the effect of the PAAm/CS/MXene hydrogel conduit on nerve regeneration, histological and ultrastructural analyses were performed at 12 weeks post-implantation. Immunofluorescence staining of cross-sectional nerve tissues showed abundant NF200-positive axons and S100-positive Schwann cells in the 600 μg/mL group (Fig. 9A). Quantitative analysis revealed that the fluorescence-positive areas of both S100 and NF200 were significantly higher in the 600 μg/mL group than in the 0 μg/mL and Blank groups, approaching the level of the autograft group (Fig. 9B and C). Given the four-channel structure of our conduit, regenerated nerve fibers grow independently within the four channels. Conventional longitudinal frozen sections cannot capture all four nerve bundles in one slice, which may lead to incomplete morphological evaluation. We therefore used whole-mount tissue clearing with 3D confocal reconstruction to visualize nerve ingrowth along the entire regenerated segment. The 3D images showed more extensive nerve fiber ingrowth and a more ordered distribution along the multi-channel cavities in the 600 μg/mL group than in the Blank and 0 μg/mL groups (Fig. 9D). TEM analysis of the ultrastructure confirmed that the 600 μg/mL group had well-organized, thick myelin sheaths surrounding myelinated axons (Fig. 9E). Consistently, the diameter of myelinated fibers and myelin sheath thickness were significantly increased, while the g-ratio was significantly decreased in the 600 μg/mL group relative to the Blank and 0 μg/mL groups (Fig. 9F–H). These findings collectively indicate that the PAAm/CS/MXene hydrogel conduit effectively promotes axonal regeneration and myelination in vivo, with regenerative metrics showing no significant statistical difference relative to the autograft group.

**Fig. 9:**
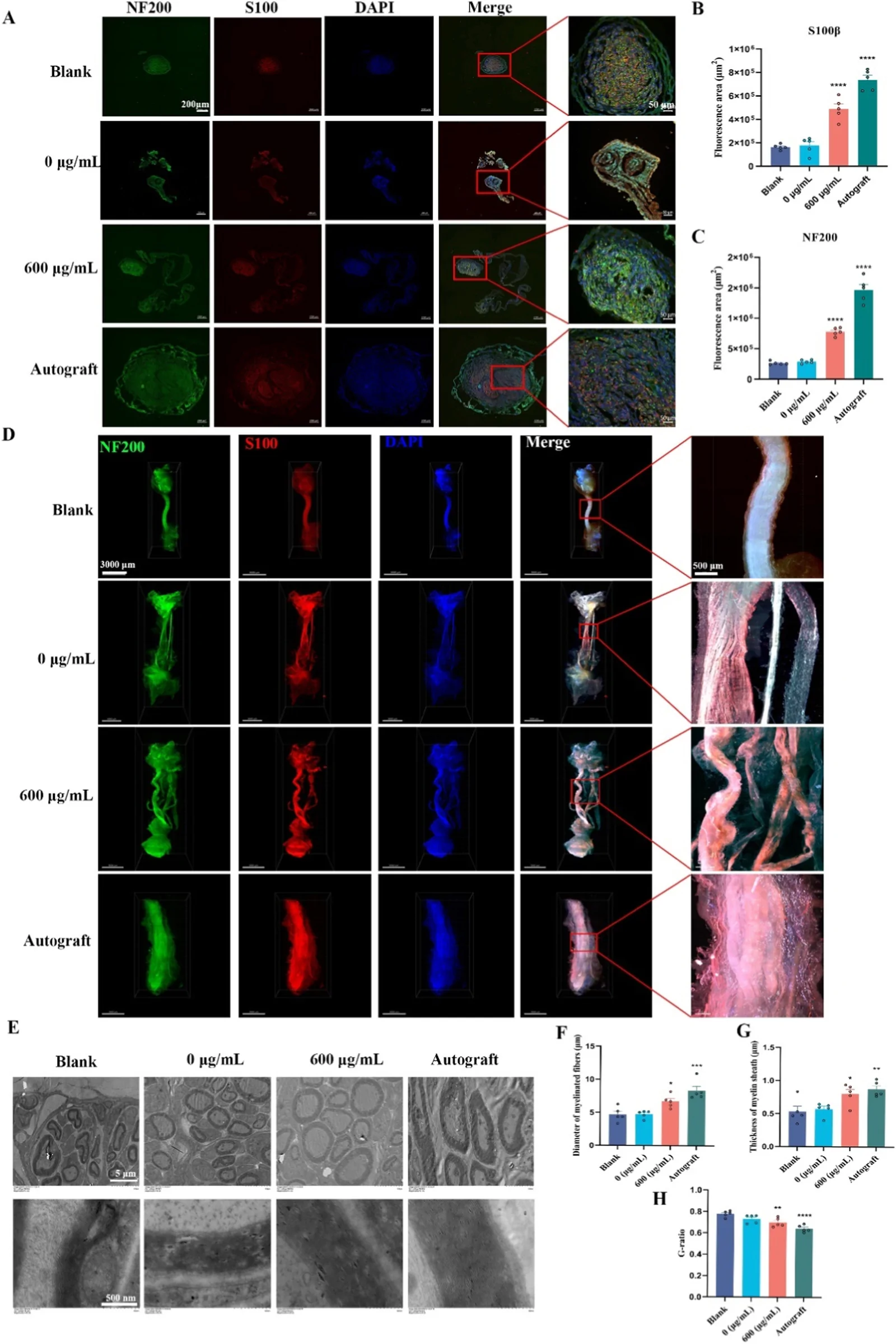
Histological and ultrastructural analysis of regenerated nerves at 12 weeks post-implantation. (A) Representative immunofluorescence staining of transverse nerve sections for NF200 (green, axons) and S100 (red, Schwann cells), with DAPI (blue) counterstaining. Scale bars: 200 μm (overview) and 50 μm (magnified views). (B-C) Quantitative analysis of the fluorescence-positive areas of S100 and NF200, respectively (n = 6, ****P < 0.0001 vs. Blank group). (D) 3D confocal imaging of whole-mount regenerated nerves, co-stained with NF200 (green) and S100 (red). Scale bars: 3000 μm (overview) and 500 μm (magnified views). (E) Transmission electron microscopy (TEM) images showing the ultrastructure of regenerated nerve fibers (upper row, scale bar: 5 μm) and myelin sheaths (lower row, scale bar: 500 nm). (F-H) Quantitative analysis of the diameter of myelinated fibers, myelin sheath thickness, and g-ratio (n = 5, *P < 0.05, **P < 0.01, ****P < 0.0001 vs. Blank group).

## 4 Discussion

The central finding of this study is that MXene nanosheets, when integrated into a PAM/CS hydrogel at a precisely tuned concentration, create a percolation conductive network that fundamentally reshapes the electrophysiological microenvironment for peripheral nerve repair. Rather than treating conductivity as a binary property—either present or absent—we show that it operates across a concentration-dependent threshold, and that crossing this threshold at 600 μg/mL triggers a disproportionately large biological response. This is not a trivial observation; it suggests that neural cells are exquisitely sensitive to the density and connectivity of conductive pathways, not merely to the presence of conductive material. MXene concentration turns out to be a multiscale control knob. At the nanoscale, it governs nanosheet dispersion and the formation of continuous percolation pathways. At the microscale, it dictates porous architecture, mechanical stiffness, and nutrient permeability. At the cellular scale, it determines whether the material presents a supportive or hostile surface.At 600 μg/mL, these scales align favorably: MXene nanosheets disperse uniformly via hydrogen bonding and electrostatic interactions with chitosan's amino and hydroxyl groups [43], forming continuous conductive networks without sacrificing the hydrogel's porous structure or swelling stability [44]. The nanosheets also serve as multifunctional physical crosslinkers and stress-dissipation centers, boosting mechanical strength while preserving compliance matched to native nerve tissue [45].

But there is a ceiling. ush the concentration too high, and van der Waals forces and hydrophobic interactions drive nanosheet aggregation. The result is a cascade of problems: collapsed pores, elevated local stiffness, hindered nutrient and oxygen exchange, and in some cases, oxidative stress and cytotoxicity [46], [47]. This is why 600 μg/mL matters—it sits at a sweet spot where conductivity, mechanical integrity, and biosafety are simultaneously satisfied. The hydrogel retains its 3D porous network, offers suitable mechanical support, and presents a cell-friendly surface, collectively providing a proper substrate for adhesion, proliferation, and differentiation of neural cells.

The cellular response bears this out. RSC96 Schwann cells and PC12 neurons—the two workhorses of peripheral nerve regeneration—both thrived on the optimized hydrogel. Schwann cells proliferated more vigorously and secreted more neurotrophic factors, while PC12 cells differentiated more readily into neuron-like phenotypes [48], [49]. These outcomes do not arise from any single property in isolation. The interconnected porous structure provides ample surface area for anchorage; the pore size allows nutrients in and pseudopods out; the conductivity mimics the bioelectrical microenvironment of peripheral nerves; and chitosan together with MXene modulates cell-surface receptor responses [50], [51]. It is the combination, not any individual factor, that drives the biological effect.

To understand how this combination works at the molecular level, we turned to transcriptomic sequencing. The data revealed abundant differentially expressed genes in both RSC96 and PC12 cells, enriched in processes spanning cell adhesion, proliferation, neuronal differentiation, and neurotrophic factor secretion. Pathway analysis pointed to two prominent signaling cascades: PI3K-Akt and MAPK [43], [48], [50].The PI3K-Akt axis is well known as a master regulator of cell survival, proliferation, and differentiation. In the context of peripheral nerve repair, it promotes Schwann cell expansion, upregulates NGF and BDNF secretion, and accelerates neuronal differentiation and axonal outgrowth [52], [53], [54]. The MAPK pathway, meanwhile, handles neurite extension, synaptogenesis, and cellular stress adaptation—processes essential for rebuilding functional neural circuits after injury [55], [56]. What our data add to this established picture is the link between material conductivity and pathway activation. The conductive PAM/CS/MXene hydrogel does not merely provide a passive substrate; it establishes a “material physical microenvironment → intracellular signaling → transcriptional reprogramming” regulatory axis. Biomimetic topography, nerve-matched mechanical compliance, and tissue-like electrical cues converge to activate PI3K-Akt and MAPK cascades, which in turn remodel gene expression to orchestrate Schwann cell proliferation, neurotrophic factor release, neuronal differentiation, and axonal maturation.This multilevel regulation ultimately manifests in the animal model as improved motor function recovery, orderly axonal remyelination, and reduced gastrocnemius muscle atrophy. Collectively, conductive MXene and bioactive chitosan establish a synergistic physical–biochemical microenvironment, and transcriptomic data provide a plausible molecular mechanism underlying this effect.

Recent studies have advanced hydrogel-based nerve conduits along several directions. Hierarchical hydrogel films with microgrooves and nanopores have been used to guide oriented neuronal growth and scavenge ROS in spinal cord injury repair, but these systems rely on topographical and biochemical cues without electrical activity [57]. Conductive nerve guidance conduits based on CNTs and ice-templated GelMA can direct neurite extension through aligned structures, yet their conductivity is not optimized around a percolation threshold, and nerve growth factor is required to achieve effective regeneration [58]. Responsive sponge nerve conduits combining PDA@BP hydrogels with aligned nanofibers have been developed to release growth factors under near-infrared stimulation, but the underlying cell–matrix mechanotransduction pathway remains unclear [59].

In contrast to these systems, the present study develops percolation-tunable conductive MXene/hydrogel multi-channel conduits. By tuning the MXene concentration around the percolation threshold, we achieve programmable control over the hydrogel's electrical conductivity. Our results further show that the conductive hydrogel regulates neural regeneration through integrin-mediated mechanotransduction, providing new mechanistic insight into how conductive hydrogels promote peripheral nerve repair. Notably, effective regeneration was achieved without exogenous stem cells, growth factors, or external stimulation.

How does this material stack up against existing options? Against non-conductive PAM/CS hydrogels, the addition of MXene introduces stable electroactivity that mimics the endogenous electric field of nerve tissue, further facilitating neural cell maturation and axonal extension [60], [61], [62]. Against carbon-based fillers like graphene and CNTs, MXene offers superior hydrophilicity, better aqueous dispersibility, and a more favorable biosafety profile [47], [62]. And against commercial nerve guidance conduits or traditional conductive polymer scaffolds, the PAM/CS/MXene system brings together an ECM-mimicking 3D porous structure, tunable mechanics, stable electroactivity, and chitosan-derived bioactivity for cell adhesion and inflammation mitigation [60], [61], [62], [63]. Notably, effective regeneration was achieved without exogenous stem cells or additional growth factors, which cuts down on immune rejection risk and simplifies manufacturing—both significant advantages for clinical translation.

From a practical standpoint, the hydrogel has several features that clinicians would appreciate. It is injectable and capable of in-situ gelation, enabling minimally invasive implantation and conformal filling of irregular nerve defects [64], [65]. The chitosan-based composition allows gradual biodegradation at a rate matched to nerve regeneration kinetics, avoiding secondary surgery for removal. And the one-pot preparation is straightforward, low-cost, and scalable—factors that matter enormously if this ever moves from bench to bedside.

That said, this study opens doors rather than closing them.Several directions look particularly promising. One is the construction of oriented microchannel conduits via 3D or 4D bioprinting to guide directional axonal growth more precisely [66], [67]. Another is the loading of neurotrophic factors or anti-inflammatory agents for sustained release, creating synergistic therapeutic effects [26], [27]. Surface functionalization with cell-adhesive peptides such as RGD or IKVAV could further boost cytocompatibility and neurite guidance [39]. And pairing the conductive hydrogel with exogenous conductive might amplify the pro-regenerative effects even further [16], [38].

Of course, there are caveats. First, the long-term in vivo biosafety and degradation behavior of MXene nanosheets—including systemic distribution, metabolic clearance, and chronic inflammatory response—warrants observation over 6–12 months in larger cohorts [6]. Second, while our rat model is informative, the repair efficacy for long-gap defects exceeding 15 mm needs validation in large-animal models with greater anatomical and physiological similarity to humans. Third, the pore microstructure in this study was characterized by SEM of freeze-dried hydrogels, which cannot provide absolute 3D bulk pore parameters because dehydration causes structural shrinkage. In future work, synchrotron X-ray micro-CT or mercury intrusion porosimetry will be used to characterize the pore architecture of hydrated hydrogel scaffolds. Finally, the regulatory mechanism of the conductive hydrogel has not been fully validated at the functional level. Our transcriptomic profiling and qPCR results show gene-expression changes, but they cannot establish whether integrin–PI3K-Akt–FAK signaling plays a causal role in hydrogel-mediated nerve regeneration. Although our data point to the involvement of this mechanotransduction axis, pharmacological inhibition or gene knockdown experiments are needed to confirm its functional contribution. In addition, Western blotting and immunofluorescence validation of PI3K-Akt and MAPK pathway activation at the protein level would help strengthen the mechanistic interpretation and clarify how these pathways are regulated. Addressing these gaps will be important for the clinical translation of this platform.

## CRediT authorship contribution statement

**Fang Liu:** Writing – review & editing, Writing – original draft, Supervision, Conceptualization. **Yixuan Zhao:** Validation, Methodology, Investigation, Formal analysis. **Yumeng Ren:** Validation, Data curation. **Chunzhen Jiang:** Validation, Methodology. **Mengjie Xu:** Validation, Methodology, Investigation, Formal analysis. **Yifan Li:** Validation, Funding acquisition. **Yingxin Wei:** Validation, Methodology. **Xiuyu Wen:** Validation, Methodology. **Zongmei Ding:** Validation. **Feng Li:** Validation. **Luzhong Zhang:** Methodology, Investigation. **Yumin Yang:** Investigation, Conceptualization. **Jianghong He:** Validation, Investigation, Funding acquisition.

## Ethics approval and consent to participate

The study received approval from the institutional review board of Nantong University. Animal Ethics Protocol ID: S20250715-109.

## Declaration of competing interest

The authors declare that they have no known competing financial interests or personal relationships that could have appeared to influence the work reported in this paper.

## Data Availability

Data will be made available on request.
