# Supplementary material for: Percolation-tunable conductive MXene–hydrogel multi-channel conduits promote integrin-mediated peripheral nerve regeneration
Source: Mater Today Bio. 2026 Nov 7;41:103822. doi: 10.1016/j.mtbio.2026.103822 (PMC13770611; doi:10.1016/j.mtbio.2026.103822)
Supplement: Supplementary file 1 [file mmc1.docx]

Fig. S1. Macroscopic photographs demonstrating the bending, pinching and shape-recovery behavior of the PAAm/CS/MXene hydrogel nerve conduit.
